# Development and Validation of a Portable EIT System for Real-Time Respiratory Monitoring

**DOI:** 10.3390/s24206642

**Published:** 2024-10-15

**Authors:** Fabian Alvarado-Arriagada, Bruno Fernández-Arroyo, Samuel Rebolledo, Esteban J. Pino

**Affiliations:** Department of Electrical Engineering, Faculty of Engineering, Universidad de Concepcion, 219 Edmundo Larenas, Concepción 4070409, Chile; fabian.alvarado@biomedica.udec.cl (F.A.-A.); bruno.fernandez@biomedica.udec.cl (B.F.-A.); srebolledo2018@udec.cl (S.R.)

**Keywords:** electrical impedance tomography, respiratory monitoring, portable medical device

## Abstract

This work contributes to the improvement of novel medical technologies for the prevention and treatment of diseases. Electrical impedance tomography (EIT) has gained attention as a valuable tool for non-invasive monitoring providing real-time insights. The purpose of this work is to develop and validate a novel portable EIT system with a small form factor for respiratory monitoring. The device uses a 16-electrode architecture with adjacent stimulation and measurement patterns, an integrated circuit current source and a single high-speed ADC operating with multiplexers to stimulate and measure across all electrodes. Tests were conducted on 25 healthy subjects who performed a pulmonary function test with a flowmeter while using the EIT device. The results showed a good performance of the device, which was able to recognize all respirations correctly, and from the EIT signals and images, correlations of 96.7% were obtained for instantaneous respiratory rate and 96.1% for tidal volume prediction. These results validate the preliminary technical feasibility of the EIT system and demonstrates its potential as a reliable tool for non-invasive respiratory assessment. The significance of this work lies in its potential to democratize advanced respiratory monitoring technologies, making them accessible to a wider population, including those in remote or underserved areas.

## 1. Introduction

One of the global goals for sustainable development is to ensure healthy lives and promote well-being for all at all ages. Non-communicable diseases such as respiratory, cardiovascular and cancer are among the main causes of morbidity and mortality in the world. Respiratory diseases, specifically, are responsible for millions of deaths, with COPD as the third leading cause of death in the world being the most deadly chronic respiratory disease, and asthma as the most common chronic disease of childhood with an increasing prevalence [1]. Early detection of risk allows patients to take preventive medical treatments to control and avoid their development. In this way, people can have access to a better quality of life and health services can save much-needed resources.

Respiratory monitoring plays a crucial role in the management and treatment of these diseases. Traditional respiratory monitoring techniques, such as spirometry, plethysmography and capnography, although effective, are often limited by the need for specialized equipment and settings [2]. These limitations pose significant challenges, particularly in the context of home care or in resource-limited settings, where the need for accessible, affordable and reliable respiratory monitoring solutions is important.

In this scenario, electrical impedance tomography (EIT) has emerged as a novel technology for respiratory monitoring. EIT is a noninvasive imaging technique that allows real-time visualization of regional lung ventilation by measuring the electrical impedance of the chest. The technique is based on the fact that different tissues and fluids in the body have different electrical conductivities [3]. By applying small electrical currents through electrodes placed around the chest and measuring the resulting voltages, EIT generates dynamic images that reflect the distribution of air in the lungs [4].

The design of the circuit electronics consists of applying current and measuring voltage; generally, multiplexers are used to distribute the current to all electrodes. Current can be applied with one or several sources and is generally a sinusoidal signal with a known magnitude and frequency [5]. This current flows through different patterns, passing through all the electrodes at least once. On the other hand, the differential voltage between electrodes is measured with a voltmeter, multiplexing across all electrodes or with several simultaneously, which reduces the measurement time in exchange for using more hardware [6].

In the late 1970s, early research on EIT began to explore its potential in pulmonary medicine, particularly its ability to provide spatial information from impedance measurements in the chest [7]. Nearly a decade later, in the mid-1980s, the first EIT devices were constructed, with the 16-electrode design emerging as a fabrication standard [8]. In recent decades, studies demonstrated the usefulness of chest EIT mainly for monitoring critical patients in intensive care units [9], although there have also been studies on cardiac perfusion and cardiac output [10,11] and chronic respiratory diseases [12,13], where it can be used as a preventive and diagnostic method for respiratory diseases, lung function assessment [14], or as an indicator to support clinical decisions in pathological cases [15].

Today, EIT faces several challenges that have limited its widespread adoption. Problems related to spatial resolution of images, lack of standardization between devices and protocols, and complexity of interpretation of impedance data, especially when used in diverse patient populations with different body compositions, have been highlighted in the literature [16].

Currently, there are different types of EIT systems, with multiple applications and numbers of electrodes. The most common are those with 16 electrodes [17], but there are also alternatives with a greater number of electrodes, reaching belts with 32 electrodes [18,19], 48 electrodes [20] and even 90 electrodes [21], for 2D and 3D reconstructions. Among the technologies that target the miniaturization and portability of devices are IC-based devices [22], fabricated in system-on-chip with CMOS technology [23,24], and wearable devices [25]. To date, five commercial EIT producers have been reported worldwide in various works [26].

This study presents the development and validation of a novel EIT-based device designed specifically for respiratory monitoring. Our focus is on addressing the high technical requirements of medical devices used for monitoring and diagnostics. The device is intended to be used in a user-friendly and non-invasive manner to provide reliable, real-time respiratory data, with the added advantage of being portable, with a small form factor, and affordable, to democratize its access, making it suitable for use in a variety of environments.

In this article, we detail the design and implementation of the EIT device, the methodologies employed for its validation, and the results of human subject trials. This work was created as an upgrade to the impedance pneumography device presented in [27].

## 2. Materials and Methods

The methodology of this work is divided into hardware and software development, and data acquisition and processing.

### 2.1. Hardware

The system uses the typical 16-electrode EIT architecture (numbered from E1 to E16) which consists of generating a known alternating current for tissue stimulation and measuring differential voltages at the resulting electrodes. This requires a current source and an ADC analog-to-digital converter in conjunction with amplifiers to measure the voltages. Analog multiplexers are also added to distribute the current to different electrodes and to select specific electrodes to measure the voltage.

**Figure 1:**
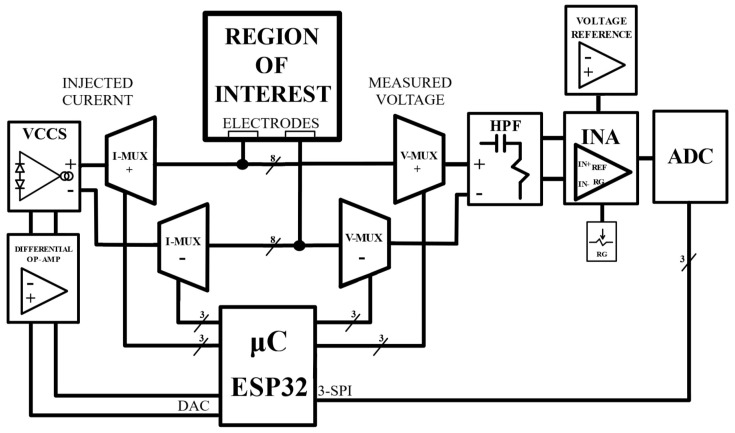
Block diagram of the EIT device.

To control the operation of all the components, an ESP32-PICO-KIT v4 module was used (Espressif Systems, Shanghai, China), which has a WiFi and Bluetooth connection, as well as multiple functions and peripherals. This model was chosen because it is a very versatile microcontroller with reduced cost and size, but with great power and connectivity, which makes it ideal for application in portable and low-power devices. A schematic of the device is shown in Figure 1, and each part is detailed below.

#### 2.1.1. Control Signal Synthesis

A control signal is required for the current source. This control signal is used to define the shape and frequency of the stimulation current. The waveform used was sinusoidal with a frequency of 50 kHz. The microcontroller has an 8-bit, 2-channel digital-to-analog converter (DAC) functional block with an integrated Cosine Waveform (CW) generator that operates independently of the main processor. The CW generator was configured on channel 1 of the DAC with the following output:

(1) $$v_{DAC}=1.65\;cos\left(2\pi \;50k\;t\right)+1.65\;\left[V\right],$$

**Figure 2:**
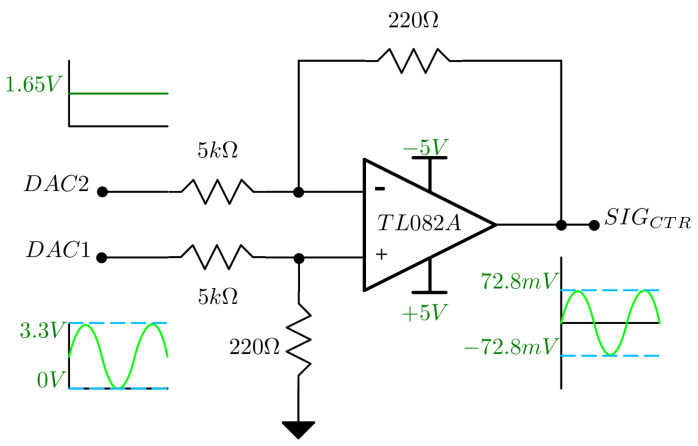
Differential circuit with Operational amplifier. DAC1 is channel 1 of the DAC, connected internally to CW generator, and DAC2 is channel 2 with a constant voltage value.

The control signal must have a zero mean value and an amplitude in the order of mV to drive the current source. Since the maximum configurable divider of the CW generator was not sufficient to reduce the signal amplitude, a differential circuit with a reference voltage equal to the offset defined as the output of channel 2 of the DAC was used to attenuate the signal. Equation (3) describes the circuit function:

(2) $$v_{REF}=1.65\;\left[V\right],$$

(3) $$v_{o}=\left(v_{DAC}-v_{REF}\right)\;\frac{220\;\Omega }{5\;k\Omega }\;\left[V\right],$$

where $v_{o}$ is the output of the differential circuit (control signal), and the attenuation factor is defined by the resistors used. Figure 2 shows the differential circuit.

#### 2.1.2. Current Source

The Operational Transconductance Amplifier (OTA) LM13700 from Texas Instruments, Dallas, TX, USA, was used to implement a fully differential current source, to ensure an adequate common mode voltage rejection. The OTA generates an output current proportional to a differential input voltage, acting as a Voltage Controlled Current Source (VCCS) [28].

**Figure 3:**
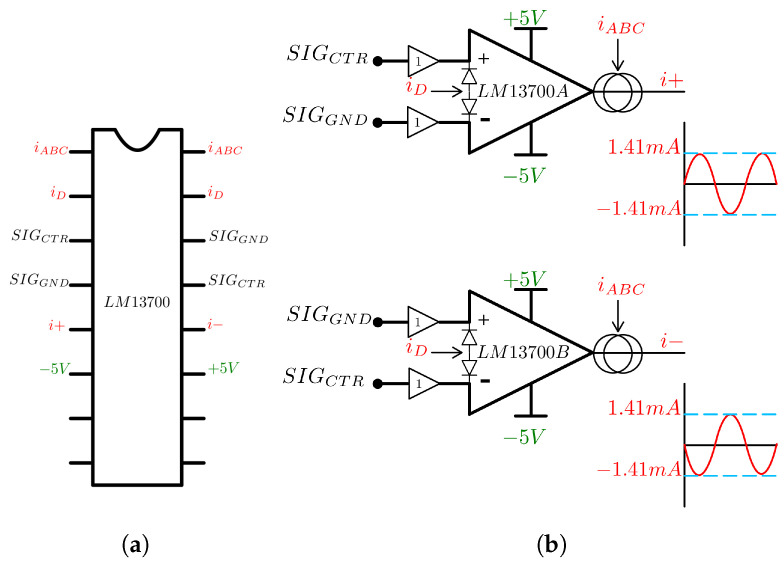
Schematics of the current source. In (**a**) the OTA connection, and in (**b**) the amplifiers with both differential outputs.

The schematic of the OTA is shown in Figure 3 with its respective pins. The control signal and a buffered 0 V signal were connected to the differential inputs inversely, to obtain two outputs of equal amplitude shifted by 180°. The amplifier bias current ($i_{ABC}$) pin was fed with a 1 ${\mathrm{mA}}_{DC}$ current and to set the gain, a potentiometer was adjusted to obtain an output of 1 ${\mathrm{mA}}_{RMS}$.

The rectifying diodes of the circuit were used with a constant direct current ($i_{D}$) to improve circuit stability, and all input signals were buffered to minimize the significant effect of the input impedance that generates an unwanted offset in the output when there is an imbalance between them. This results in a fully differential current source of 1 ${\mathrm{mA}}_{RMS}$ at 50 kHz. The LM13700 from Texas Instrument, Dallas, TX, USA, has the advantage of having two amplifiers in the same package, saving hardware space.

#### 2.1.3. Multiplexers

Stimulation current from the source is applied to the 16 adjacent electrode pairs (E1–E2, E2–E3, …, E15–E16, E16–E1) sequentially. Since only one source is available, a system of two 1:8 analog multiplexers was implemented to perform all combinations. Each current source output was connected to common inputs, while the even electrodes were connected to the outputs of one multiplexer and the odd electrodes to the outputs of the other.

The multiplexer circuit was replicated for the selection of electrode pairs for voltage measurement. While current is applied to one pair, the differential voltage is measured at the other pairs of non-conducting electrodes. The connection is identical, but in this case, the electrodes act as inputs, and the selected electrode pair as common outputs. These common outputs are connected to a high-impedance circuit, and the unselected channels of the multiplexer have a high impedance due to their internal operation; therefore, non-conducting electrodes do not interfere with the stimulation current path.

**Figure 4:**
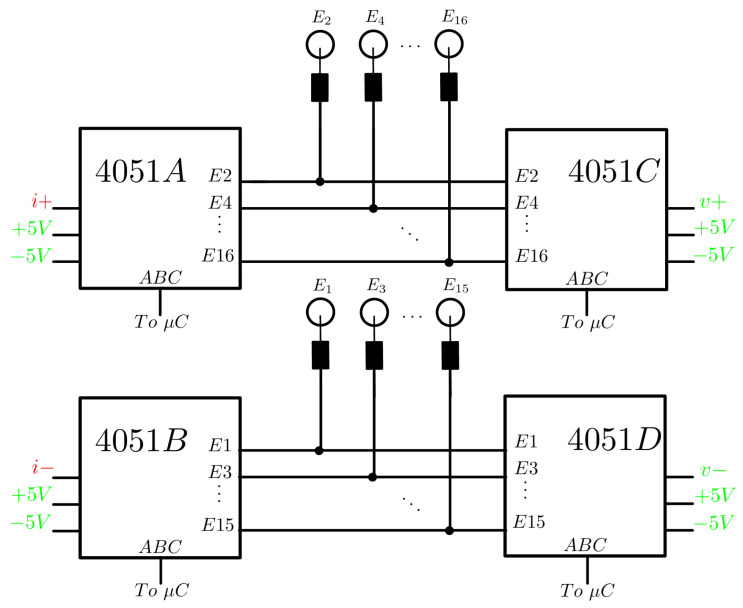
Multiplexer connection diagram. *A* and *B* are the stimulation current multiplexers with i+ and i− as inputs coming from the current source. *C* and *D* are the voltage multiplexers with v+ and v− as differential voltage outputs. The 12 ABC selectors are connected to the microcontroller on independent digital outputs.

The model used was the CD4051. The three selectors of each multiplexer were connected directly to the digital outputs of the microcontroller. The connection diagram can be seen in Figure 4. To connect the electrodes, 3.5 mm audio jack connectors were used, which allowed the plugging of three-snap lead electrode cables.

#### 2.1.4. Voltage Amplification

The differential voltages resulting from the non-conductive electrodes are in the order of microvolts, for this reason, an instrumentation amplifier model INA128 (Texas Instrument, Dallas, TX, USA) was used to amplify the voltage. These amplifiers are characterized by having a high common-mode rejection ratio (CMRR). Two resistors of equal value were connected in series to the gain pins to adjust the gain ($G_{INA}$) value according to the following equation:

(4) $$G_{INA}=1+\frac{50\;k\Omega }{R_{G}}\;=1+\frac{50\;k\Omega }{2\;(250\;\Omega )}\;=101.$$

**Figure 5:**
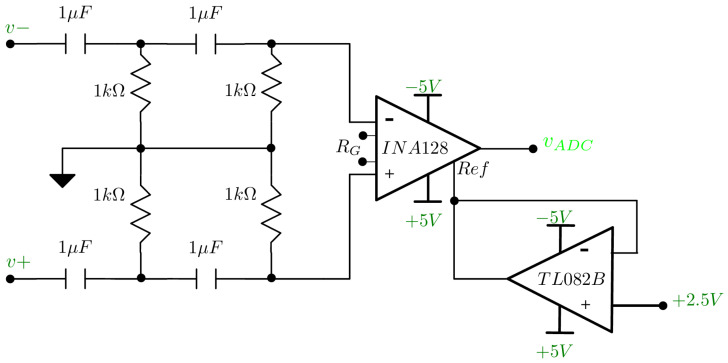
Connection of the INA with the floating $R_{G}$ pins, the v− and v+ inputs on the high-pass filters and the reference voltage follower.

The output of the voltage selector multiplexer of the even electrodes (v+) was connected to the non-inverting input, while the output of the multiplexer of the odd electrodes (v−) was connected to the inverting input. In addition, second-order passive high-pass filters were implemented at both inputs to reduce the common-mode voltage by eliminating any low-frequency components present in the selected electrode pairs. The cutoff frequency is calculated from Equation (5):

(5) $$f_{c}=\frac{1}{2\pi \sqrt{R_{1}C_{1}R_{2}C_{2}}}\;\left[\mathrm{Hz}\right]=\frac{1}{2\pi \sqrt{{\left(1\;k\Omega \right)}^{2}\;{\left(1\;\mu F\right)}^{2}}}\;\left[\mathrm{Hz}\right]=159.15\;\left[\mathrm{Hz}\right],$$

the reference voltage pin of the amplifier was connected to a 2.5 V obtained from a buffered voltage divider. This voltage was used to center the amplifier output to the ADC operating ranges. Figure 5 shows the instrumentation amplifier connection.

#### 2.1.5. Reference Electrode

A modified Driven Right Leg (DRL) circuit was implemented for the reference electrode. The DRL is a circuit added to biological signal amplifiers to reduce electromagnetic interference and common-mode voltage [29]. This circuit is frequently used in point-of-care devices [30].

**Figure 6:**
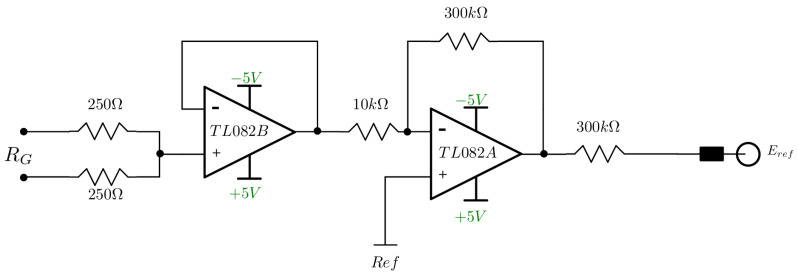
Implemented DRL circuit, connected to the $R_{G}$ pins of the INA. In this case, the gain resistor of 500Ω is divided into two resistors of 250Ω each.

The circuit modification was made according to the manufacturer to be implemented in conjunction with the INA128, which does not allow manipulating the internal resistors to make the original DRL, as shown in Figure 6. The influence of the reference electrode is discussed in [31].

#### 2.1.6. Measurement and Conversion

Considering the frequency of the signals obtained, a converter with a sampling frequency sufficient to correctly digitize the signals and a high resolution to reduce quantification errors is needed. The Digilent PmodAD1 (Digilent, Pullman, WA, USA), a two channel 12-bit ADC, was used to measure the differential voltages of the INA output.

Both the reference voltage and the operating voltage of the ADC were set to 5 V, defining the resolution:

(6) $$ADC_{RES}=\frac{5\;V}{2^{12}\;\mathrm{bit}}=1.22\;\left[\mathrm{mV}/\mathrm{bit}\right].$$

**Figure 7:**
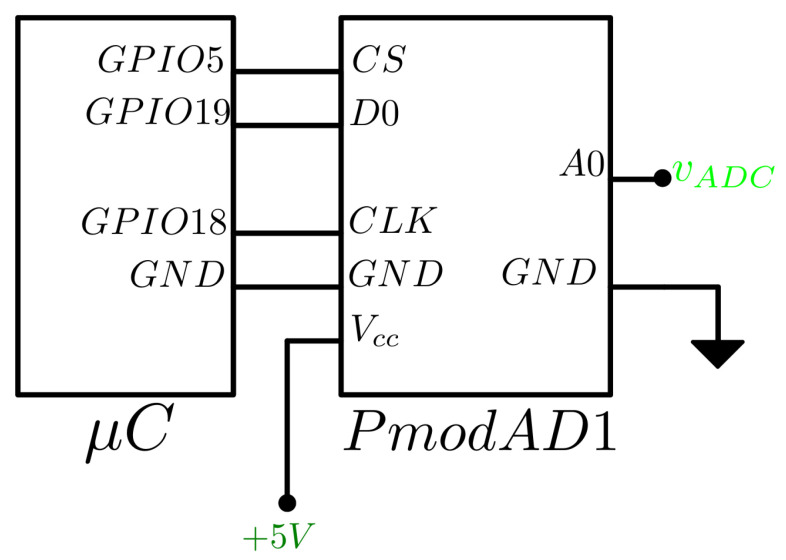
Connection between the ADC and the microcontroller.

The configuration of the converter was conducted through the microcontroller via SPI communication. The connection diagram is shown in Figure 7. This type of connection only uses three lines: CS, SCLK, and MISO, while MOSI is not used.

#### 2.1.7. Energization

**Figure 8:**
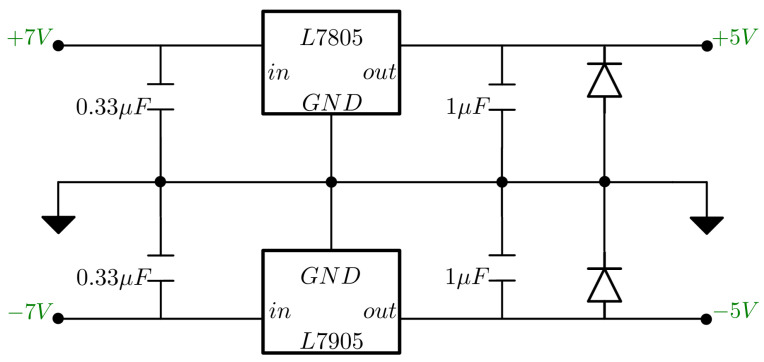
Circuit with positive and negative voltage regulators.

All analog components of the circuit were energized with ±5 V. These voltages were obtained from a series-configured DC power supply connected to a 7805 voltage regulator circuit for the positive voltage and 7905 for the negative voltage. Bypass capacitors and protection diodes were added in both regulator circuits for the cases of over-voltage spikes, input short circuits, or reverse current flow. The circuit is shown in Figure 8. The ADC was powered with 5 V from the voltage regulator, and the microcontroller with 5 V from the USB connection.

For human safety, the current reference voltage used is generated by the microcontroller at the maximum possible value (3.3 V) and then attenuated using board components that cannot be modified. Also, the microcontroller and integrated circuits (IC) supply voltage is limited by a voltage regulator. This way, we minimize possible points of failure that may result in an increase in the stimulus current.

### 2.2. Software

The operation of the EIT system is mainly handled by the microcontroller, as it is responsible for the correct flow of the overall program. However, the data acquired by the device are not stored in its memory, due to the large volume of data. These are sent to a computer via USB, where they are stored for further processing in dedicated software.

#### 2.2.1. Loop Initialization

The goal of the microcontroller is to ensure the correct execution of the Sheffield protocol [8], the most common pattern of stimulation and measurement. This consists of placing equidistant electrodes in a single plane with adjacent current stimulation and differential voltage measurement patterns. The adjacent configuration is chosen because it offers a better signal-to-noise ratio (SNR) for the current magnitudes used ($1\;{\mathrm{mA}}_{RMS}$), improved image reconstructions and estimation of clinical parameters [32]. In this case, there are 16 different current patterns. Then, for each current pattern, 13 differential voltage measurements are performed, since pairs with current-carrying electrodes are excluded. The following equation indicates the number of measurements to generate a single frame of EIT data:

(7) $$N_{m}=N_{E}\;\left(N_{E}-3\right)=16\;\left(13\right)=208\;\left[\mathrm{meas}.\right],$$

with $N_{m}$ the number of measurements and $N_{E}$ the number of electrodes.

To program the ESP32-PICO module, Arduino IDE was used with the ESP32 processor compatibility package from Espressif, available online. Libraries were used to enable the integrated DAC, edit the CW generator registers, and establish the communication interface with the ADC. Serial communication at 921,600 baud per second was activated for code debugging and data transmission.

A function sets the 12 multiplexer selectors to digital output mode, and then the channels for the first current pattern and the first differential voltage measurement pattern are enabled (current on E1 and E2, voltage between E3 and E4). The combinations of the multiplexer selectors with the electrodes to make the 16 adjacent combinations of stimulation are detailed in Table 1.

The combinations are similar for the differential voltage multiplexers *C* and *D*. The multiplexer outputs in Table 1 do not correspond to the IC numbering, as they were coded for convenience depending on the orientation of the multiplexer on the PCB.

#### 2.2.2. Voltage Measurement

The differential voltage signal $v_{ADC}$ of the INA is sampled with the ADC at a 350 kS/s, taking 64 samples without interruptions, which corresponds to approximately seven cycles of the signal. Then the next measurement is prepared to be sampled, so the voltage multiplexers switch to the next pair. Conditions are imposed to select the 208 combinations correctly.

At each switch, a settling time of 2 ms is given for the voltage signal to stabilize so that it can be sampled correctly. This behavior has been documented in previous work using the same multiplexer model [33]. The time was chosen for convenience to achieve a frame rate of 2 fps.

After the multiplexer switch, the samples are stored locally in an array of 64 unsigned integers. Data are composed of the voltage measured by the ADC in the 12 least significant bits, and the number of the voltage pair in the 4 most significant bits. The data are separated into two bytes using masks and bit operations, to be sent via serial communication to the PC.

#### 2.2.3. Data Preprocessing

**Figure 9:**
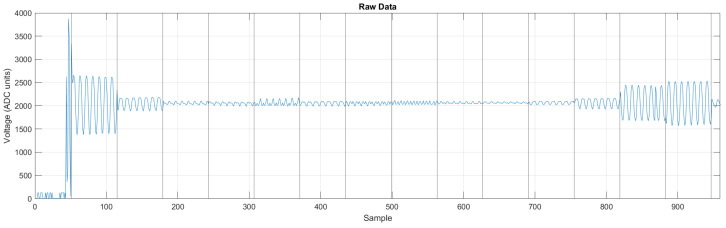
Decoded raw voltage signal. Vertical lines separate the measurements, the first data are discarded.

The raw voltage data are decoded into ordered structures. The binary file with the raw voltage data is loaded into Matlab, to clean the data. Figure 9 shows a raw signal without processing. This signal is a one-dimensional vector and has invalid data at the beginning and then the voltage data are digitized by the ADC, with each measurement following the next.

These data are reorganized into a three-dimensional matrix with size 64×208×F, where 64 corresponds to the samples in each measurement, 208 measurements per frame, and *F* number of frames. For this, the reshape function is used with the raw signal clipped with a length multiple of 64×208.

**Figure 10:**
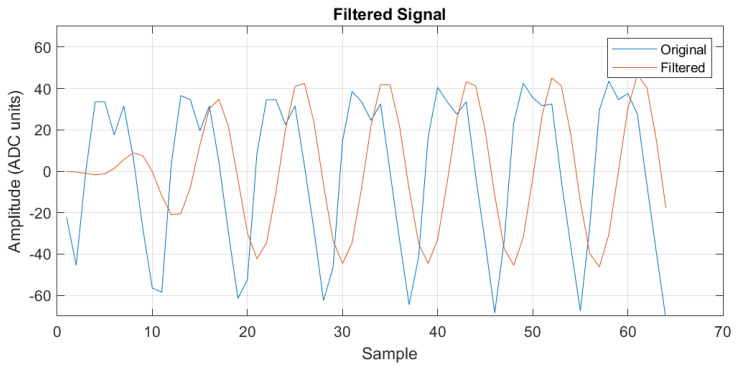
Raw and filtered differential voltage signal.

A bandpass filter is applied to the obtained signals to remove the mean value and maintain the frequency of interest. A filtered signal is shown in Figure 10.

**Figure 11:**
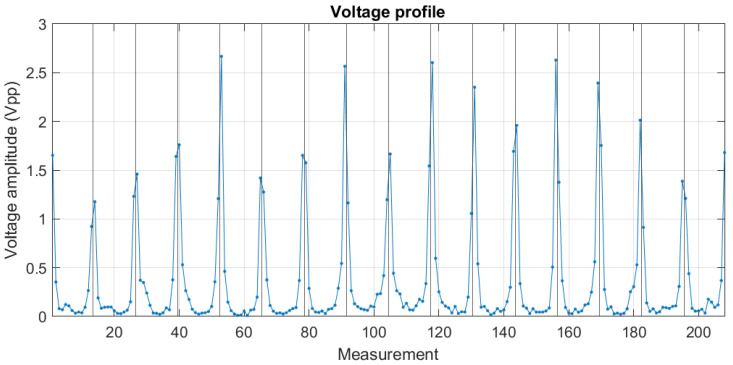
Voltage profile of a frame. The vertical lines separate the stimulation current patterns.

**Figure 12:**
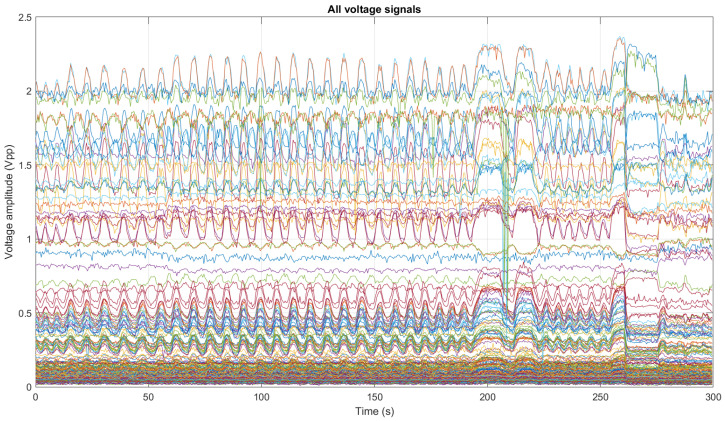
Voltage measurements for a subject with normal breathing and respiratory maneuvers at times 180 s and 250 s.

For each of the filtered signals, the peak-to-peak voltage is calculated by subtracting maximum and minimum values, forming a new 208×F dimension voltage matrix. By plotting a column of the voltage matrix, the voltage profile graph is obtained. This graph has a characteristic U-shape. An example of a voltage profile of a heterogeneous medium is shown in Figure 11. On the other hand, when several rows of the voltage matrix are plotted, the variation in these measurements over time is observed. Figure 12 shows the 208 measurements in one subject.

### 2.3. Validation

To validate the correct operation of the device, a series of tests were carried out with the participation of healthy volunteers.

The work was submitted and approved by the university’s IRB for testing on human subjects, with approval code CEBB 1304-2022. Participants had to be of either sex, aged between 18 and 60 years, and should not have a history of any respiratory disease. Participation was voluntary and 27 subjects were selected.

**Figure 13:**
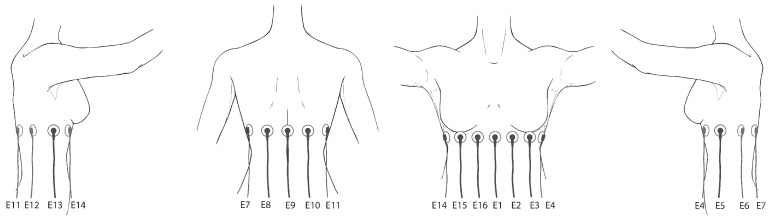
Electrode placement for female subjects. From (**left**) to (**right**): right lateral view, posterior view, anterior view, left lateral view.

The protocol developed for the test consists of obtaining informed consent from the participants, data collection with a survey, and pulmonary function simultaneous testing with the EIT device and a flowmeter. To prepare for the test, 16 contact electrodes are attached to the skin in the form of a ring around the chest, equidistant at the level of the fifth intercostal space, as shown in Figure 13, the reference electrode is placed in the abdomen. To achieve good contact, the area was cleaned using sanitary alcohol.

The test consists of two stages in which the subject must breathe through a flowmeter with the nose clamped and the EIT device attached: in the first stage, the subject must breathe normally for a period of 3 min, to define a baseline of their breathing. In the second stage, two respiratory maneuvers are performed, which require the volunteer’s cooperation. The first maneuver is a forced inspiration, where the subject breathes in the maximum volume of air possible in their lungs, the second maneuver is a forced expiration, where the subject again breathes into the maximum capacity and then blows out forcefully, exhaling as much air as possible. The registration ends automatically in 5 min.

To record the flow data, Biopac SS11LA, which is a portable air flow transducer designed for human use, was used in conjunction with the MP35 Data Acquisition Unit with the parameters of the Table 2.

The accessories used with the flowmeter are the antibacterial filter and the mouthpiece, to be placed between the subject and the sensor, the clamp goes on the nose to avoid nasal breathing. At the time of the test, the subject is required to remain seated with a straight back and hold the sensor with the accessories straight, the nose clamped and the mouthpiece inserted in the mouth so that no air escapes.

The subject is prepared by breathing through the mouthpiece and the flowmeter and EIT device recordings are started simultaneously. After 5 min, they are stopped and the files generated are saved in a folder properly identified.

### 2.4. Data Processing

Processing in Matlab was performed to detect respiration and estimate respiratory volumes in pulmonary function tests; 25 of the 27 subjects were used due to the loss of information caused by poor acquisition in two records.

**Figure 14:**
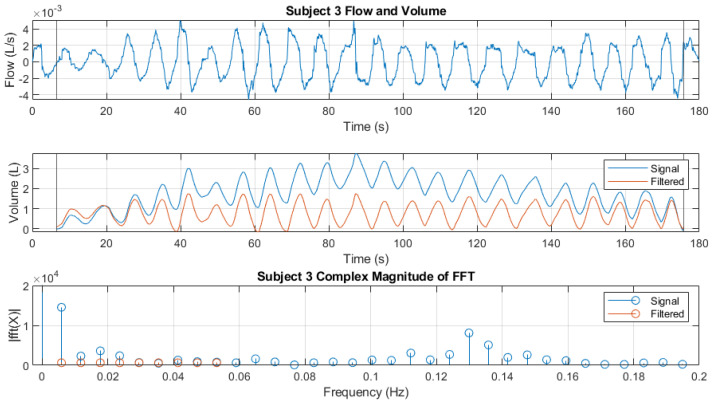
Filtered volume signal. (**Top**): flow signal with integration range limits on the x-axis, (**middle**): the volume signal and the filter output, and (**below**): the frequency components of interest of the volume signal and the applied filter.

The flow signals were manually reviewed to note peaks and valleys. For this, the findpeaks function was used for both local maxima and minima. To calculate the volume, the flow signals were calibrated with a calibration syringe of the flowmeter and then Equation (8) was applied:

(8) $$V\left(x\right)=\int_{p1}^{p2}f\left(x\right)\;dx\;,$$

with *V* volume, *f* flow, *x* samples, and points p1 and p2 defined from the index of the peaks and valleys of the flow signal according to Equations (9) and (10):

(9) $$p1=\left\lfloor \frac{x_{valley}\left(1\right)+x_{peak}\left(1\right)}{2}\right\rceil ,$$

(10) $$p2=\left\lfloor \frac{x_{valley}\left(end\right)+x_{peak}\left(end\right)}{2}\right\rceil ,$$

with $x_{valley}\left(1\right)<x_{peak}\left(1\right)$ and $x_{valley}\left(end\right)<x_{peak}\left(end\right)$. These points were chosen so as not to vertically shift the volume signal. A filter was applied to them to reduce the amplitude of the frequency components between 0 and 0.1 Hz. These components are produced by the orientation change of the sensor and generate a slight oscillation during the whole recording. However, this oscillation has a great influence on the signals obtained from the integral, since it accumulates. An example is shown in Figure 14. Finally, the manual annotation of peaks and valleys was repeated, this time for each of the volume signals.

To decide whether the device was capable of detecting respirations correctly, the voltage signals were compared with the volume signals. Each voltage signal, originally sampled at $fs_{EIT}=2\;\mathrm{Hz}$, is first interpolated by a factor of 100 to match the frame rate of the flowmeter ($fs_{flow}=200\;\mathrm{Hz}$), and then clipped between points p1 and p2.

**Figure 15:**
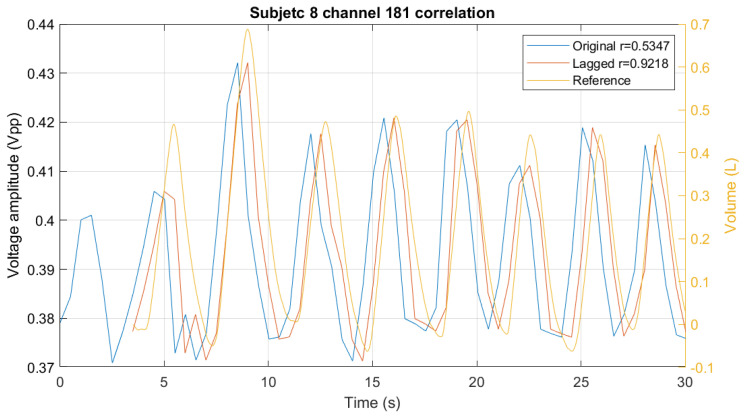
Correlation between voltage signal and volume signal (Reference). The legend shows the Pearson correlation coefficient *r* for the original clipped signal and for the shifted signal.

To compare, the cross-correlation between the two signals is calculated. The maximum correlation is attained when the voltage signal is shifted according to the cross-correlation result, as shown in Figure 15.

The process to find maximum correlation is repeated for the images generated with the EIDORS version 3.11 reconstruction software [34]. In this case, the forward model struct is configured using *mk_common_model(’i2t1’,16)*, with mesh density (a–j), model dimension (2D), chest slice (t1), and number of electrodes (16) as arguments to generate the chest model in EIDORS.

For reconstructions, a modification of the Sheffield protocol was used: in the first stimulation pattern (E1–E2), current flows from electrode E1 and leaves through electrode E2, and the differential voltages are measured in the following order: between electrodes E3 and E4, then E5 and E4, up to E15 and E16, positive and negative, respectively. While in the second stimulation pattern (E3–E2), the current still leaves from the electrode E2, but flows in through the electrode E3, and the differential voltages are measured from E5 and E4, to the E1 and E16. This difference is generated by the configuration of the current source with the multiplexers. For each current pattern a *stim_pattern* structure is generated, with 16 elements. Some examples are shown in Table 3.

This modification is also applied to each measurement pattern matrices *meas_pattern*, and the model is completed with the parameters: difference reconstruction type, and 1mA stimulation. Correlation is calculated between volume signals and the interpolated pixel values in time.

For volume estimation, the tidal volume (TV) of each respiratory cycle was calculated considering the amplitude in inspiration, i.e., between a valley and the following peak. A linear regression is used to predict TV from the voltage signals of each subject, and the correlation between the TV vector of observed and predicted data is also calculated.

## 3. Results

The main results obtained for the development of the EIT device and validation tests are detailed as follows.

### 3.1. EIT Device

**Figure 16:**
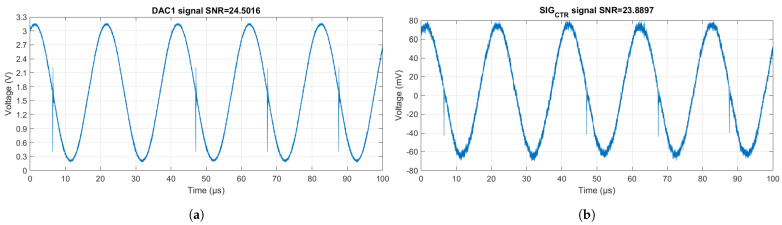
Current source signals. (**a**) Output of the ESP32 CW generator with amplitude 3.3 Vpp, and offset 1.65 V. (**b**) Output of the differential circuit with amplitude 72.8 mVp, and offset 0 V. Graphs produced by oscilloscope data. SNR: signal-to-noise ratio.

The synthesis of the control signal meets the requirements of the OTA to generate the stimulation current. Figure 16 characterizes the main signals.

The vertical line in each cycle of the generated signal is produced by the microcontroller and varies from model to model. However, this high frequency noise does not affect the voltage signals, because the sampled signals are filtered digitally in data preprocessing with a bandpass filter centered at 50 kHz.

With these signals and enabling the rectifier diodes of the OTA, an output distortion of less than 0.2% is achieved, according to the manufacturer’s information.

By adjusting the gain current, a stimulation current output of 1 ${\mathrm{mA}}_{RMS}$ with a sinusoidal shape and frequency of 50 kHz is generated. The current tolerates loads up to 10 kΩ, which is limited by the supply voltage of the circuit (±5 V). The output impedance is inversely proportional to the current gain, in this case, a 1.4 MΩ was achieved. For the voltage amplification, the instrumentation amplifier has a CMRR of 125 dB for the selected gain.

**Figure 17:**
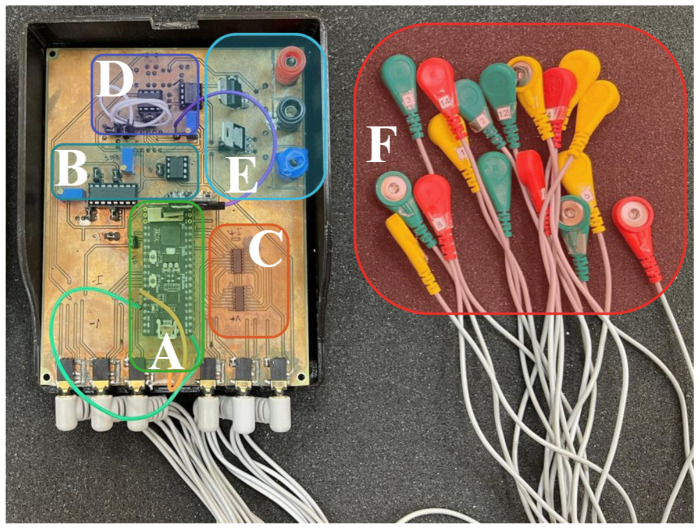
EIT device photo showing: (**A**) microcontroller unit, (**B**) voltage-controlled current source, (**C**) multiplexers stage, (**D**) voltage amplification and filters, (**E**) power circuitry, and (**F**) electrode connectors.

All the hardware was assembled on a PCB with dimensions 10 cm × 8 cm. Figure 17 shows the PCB assembled with the electrode cables and power cables. Plastic packaging for the circuit was also designed using 3D printing.

### 3.2. Subject Tests

A total of 25 subjects were selected for the experiment, of which 13 were male (52%) and 12 were female (48%). Age, height, weight and chest circumference are summarized in Table 4.

**Figure 18:**
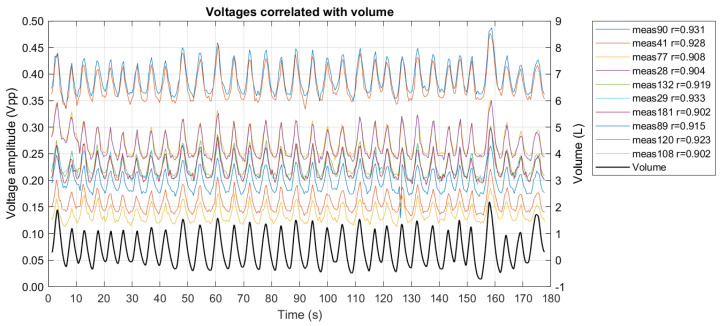
Voltages with highest correlation with volume for a subject. The volume signal is shown in black (right axis), while the voltage signals are shown in color (left axis). In the legend, the measurements are shown with their *r* coefficient.

The first three minutes of the recording were then analyzed to estimate respiration. An example of the similarity between the voltage signals with the volume signal in a subject is shown in Figure 18, taking the 10 measurements with the highest correlation coefficient.

**Figure 19:**
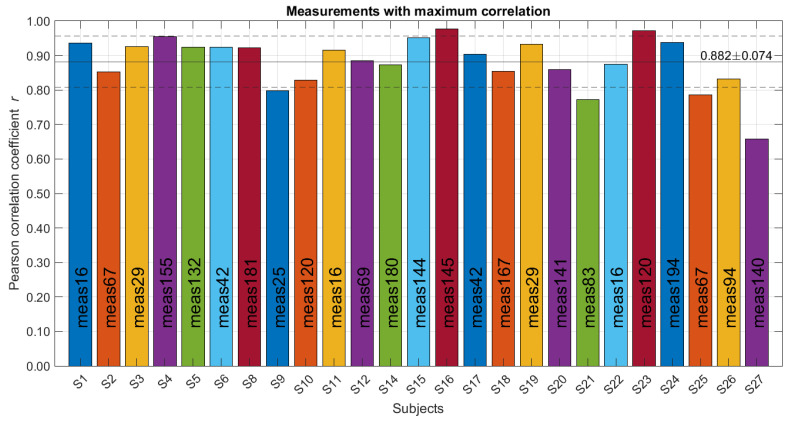
Measurements with the highest correlation per subject. On the x-axis are the 25 subjects, and each bar identifies the measurement with the highest correlation. The horizontal lines indicate mean and standard deviation.

Selecting the voltage signal with the highest correlation with volume for each subject results in a Pearson correlation coefficient *r* mean and standard deviation of the following:

(11) $$\bar{r}=0.882\;\pm \;0.074,$$

the details of each subject can be seen in Figure 19.

**Figure 20:**
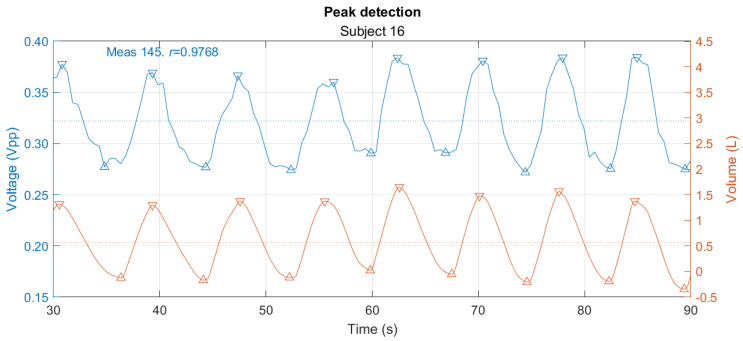
Peak detection for voltage and volume. On the left axis is the voltage signal with the highest correlation and on the right axis is the volume signal. The marks with ∇ correspond to peaks and with Δ to valleys.

**Figure 21:**
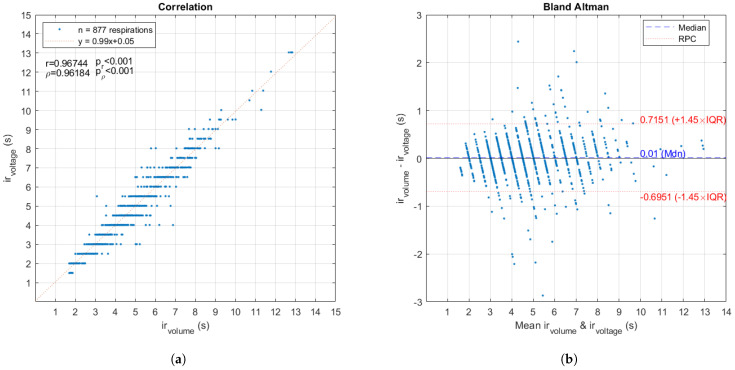
Comparison of instantaneous respiratory rate between volume signal and voltage signal for the 25 subjects. (**a**) Plot of correlation between both variables with its equation, Pearson *r* and Spearman ρ correlation coefficients, with their respective *p*-values. (**b**) Bland–Altman plot of mean between both variables vs. their difference, the median and the limits of conformity defined by the reproducibility coefficient (RPC) and interquartile range (IQR) with the formula RPC=1.45 IQR=1.45(0.0543).

After selecting the channel with the highest correlation for each subject, the algorithm for detecting peaks and valleys in the signals is applied and the indexes are obtained as shown in Figure 20. The peaks of the signals are used to calculate the respiratory rate, defining a breath as the cycle from one peak to the next. For both signals, the instantaneous respiratory rate vector is calculated using *a diff* function on the peak time vector, to obtain the duration of each respiration in seconds. Comparing the results obtained with the volume and voltage signal annotations for the instantaneous respiratory rate of the 25 subjects gives the correlation and Bland–Altman plots (Figure 21). In cases where the instantaneous respiratory rate vectors for the same subject were of different lengths, the longer vector was truncated to the value of the shorter one. For Bland–Altman, a non-parametric analysis was made.

**Figure 22:**
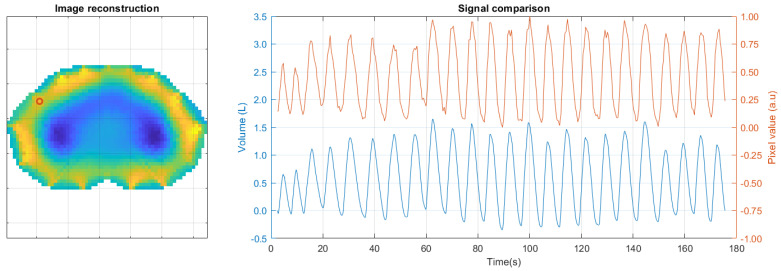
Image reconstruction. On the left the image shows a selected pixel at position (11, 25). On the right, the plot shows the variations of the selected pixel over time across all frames (**top**), along with the volume signal as a reference (**bottom**).

The processing to compare the signals was repeated with the images obtained from the reconstruction, such as the example in Figure 22. In this case, the pixel values of the image reconstructions were used.

**Figure 23:**
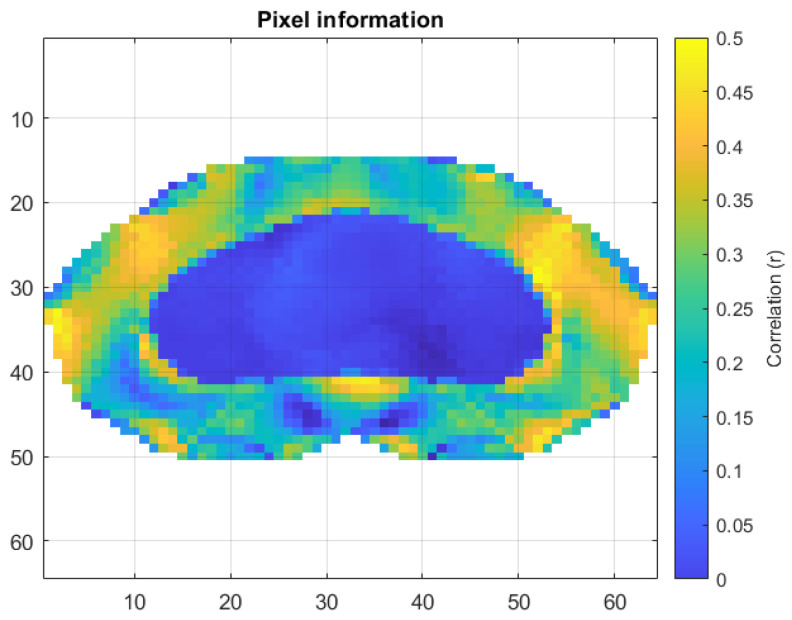
Average correlation of pixels with volume for the 25 subjects.

The pixels with the highest correlation with the voltage signal for each subject were selected to annotate the peaks and define the respirations. The average of the pixels that provided the most information to describe respiration is shown in Figure 23.

When calculating the correlations of the instantaneous respiratory rate with the pixel values of the images, slightly lower results were obtained than for the voltage signals. The results of both approaches are summarized in Table 5.

**Figure 24:**
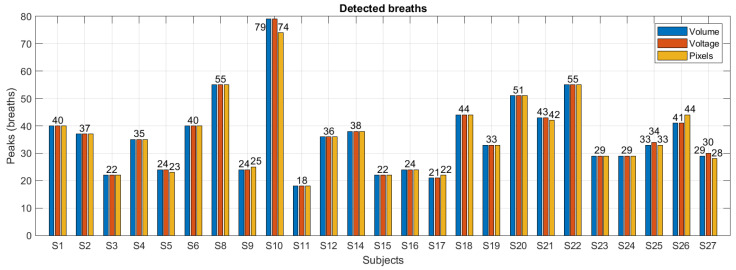
Breaths detected in the three minutes of recording with normal breathing for all subjects with volume annotations in blue, voltage signal in orange and pixel value in yellow.

On the other hand, breaths detected for each subject with the annotations of the volume signal for the voltage signals and pixel values of the images are shown in Figure 24. With these results the confusion matrix is calculated in Table 6. While with the voltage signals all breaths were recognized, with the pixel values seven respirations were missing.

With the values in Table 6, sensitivity (Se), precision (Pr) and F1-score can be calculated with Equations (12)–(14), respectively:

(12) $$Se=\frac{TP}{TP+FN}\;,\;Se_{v}=1\;,\;Se_{px}=0.9922\;,$$

(13) $$Pr=\frac{TP}{TP+FP}\;,\;Pr_{v}=0.9978\;,\;Pr_{px}=0.9944\;,$$

(14) $$F1=2\frac{Se\cdot Pr}{Se+Pr}\;,\;F1_{v}=0.9989\;,\;F1_{px}=0.9949\;,$$

where, TP true positives: breaths present in the volume signal and recognized by the method; FN false negatives: breaths present in the volume signal but not recognized by the method; FP false positives: breaths that are not in the volume signal and are recognized by the method; *v* subindex is for metrics calculated for voltage signals; px subindex is for pixel values.

**Figure 25:**
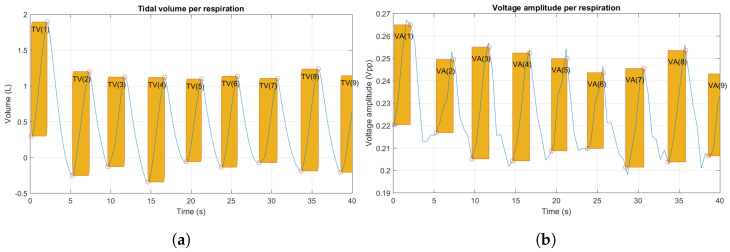
(**a**) Calculation of TV per breath for the volume signal and (**b**) amplitude corresponding to the same breath for the voltage signal.

Finally, for volume estimation predictions were calculated using the equation of linear regression, fitting the TV vector of each subject (one inspiration volume per breath calculated between a valley and the next peak) and the amplitude of the voltage signal of the same breath, using the measurement that best describes the respiration in each case, and adding point (0, 0) as a condition for adjustment, an example is shown in Figure 25.

With the regression coefficients obtained for each subject, a volume prediction is estimated, with the amplitudes of the voltage vector as input, and the correlation between the predictions and the volume is calculated. Finally, the correlation value obtained is weighted by the number of breaths of each subject. On average, the Pearson correlation coefficient *r* and Spearman correlation coefficient ρ is as follows:

(15) $$\bar{r}=0.96115\;,$$

(16) $$\bar{\rho }=0.94627\;,$$

**Figure 26:**
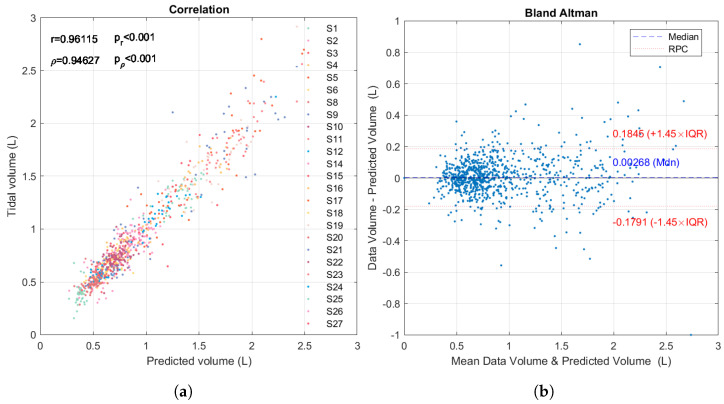
Comparison of TV between volume predictions from EIT voltage and volume signals from flowmeter for the 25 subjects. (**a**) Plot of correlation between both variables, Pearson *r* and Spearman ρ correlation coefficients, with their respective *p*-values. (**b**) Bland–Altman plot with median and limits of conformity.

Figure 26 shows the correlation and Bland–Altman plots for all subjects. Table 7 shows the details of the volume prediction results for all subjects.

## 4. Discussion

This work presents the development and validation of a portable EIT device for respiratory monitoring, from its design to the analysis of the data obtained in the tests.

Our findings show that this EIT system supports respiratory monitoring, offering a new tool to improve patient follow-up in both clinical and home settings, with seamless detection of respirations and high correlations for respiration rate and tidal volume.

The EIT system uses a 16-electrode architecture, with stimulation current generation and differential voltage measurement on the other electrodes. The use of the integrated DAC for control signal synthesis offers the advantage of space saving. The disadvantage is that once it is set up, it synthesizes the signal without stopping and there is no way to access an indicator that marks the start of a cycle. This causes the differential voltage measurements to be non-phase sensitive.

The choice of OTA was made on the basis of miniaturization, seeking as small a form factor as possible, over other options that required more circuitry. In this case, the current source consisted of a single packaged circuit, which simplified the design. The advantages of the OTA are its compact form, which has two amplifiers in a single IC, the ease of implementation and the stability of the signals. The disadvantage is its minimum operating voltage of single 9 V or dual ±4.5 V, which defines a voltage level for the entire circuit, being relatively high for a portable device. In addition, to satisfy the input voltage range limitation it was necessary to attenuate the DAC signal using the differential circuit.

The main advantages of the multiplexers are the reduction of current sources, filters and voltage amplifiers. In this way, each electrode can be connected to the source and to the amplifier. Disadvantages such as the high settling time of the selected channel and the high ON resistance are related to the multiplexer model used.

The INA performed well; however, it was necessary to filter the inputs because the high gain of the amplifier makes it very sensitive to noise. With high-pass filters, the DC component of the voltage is removed, preventing the amplifier output from saturating, while low-pass filters attenuate the high-frequency noise. In this way, the ADC can correctly digitize the filtered and amplified voltage, with minimal noise.

In the case of the EIT device, it worked correctly in all registers. The data discarded were due to difficulties in the use of the flowmeter, such as sudden movements or changes in orientation and inclination that introduced noise. Also, situations in which the subjects interrupted the recording for a moment due to discomfort (difficulty in breathing or abundant salivation) caused a discontinuity in the flow signal and an accumulation of errors that significantly displaced the volume signals.

The correlation of all voltage signals with the volume signals for each subject shows that the measurements with the highest correlation were numbers 107, 120, 108, 89, and 94 (numbered from 1 to 208). These measurements have in common the fact that the voltages are measured at a distance of three to four electrodes from the current electrodes. Anatomically, this also corresponds to a current injection from the back of the thorax and a measurement on the side of the subject (i.e., electrodes E3, E4, E5 on the right and E13, E14, E15 on the left). The measurements that have the highest dynamic range are those measured immediately next to the current application; however, these measurements have worse performance in identifying respiration.

Note, 100% of the respirations are detected from the measurement with the highest correlation to the volume signal. Although the selected voltage measurement is subject-dependent, the results show a consistent selection of the side electrodes, as mentioned in the previous paragraph.

Compared to the strong results provided by [14], our work uses a higher number of subjects (*n* = 25 vs. *n* = 14) and achieves a higher correlation in the prediction of TV (96% vs. 89%). When comparing the performance of the voltage signals with the functional images, the evidence shows that the signals are slightly better (96% vs. 92%). Still, the reconstructed images provide a very good estimator, and these can be improved by adjusting the reconstruction parameters. On the other hand, Bland–Altman plots show that the EIT device is appropriate to measure respiratory frequency and TV compared with flowmeter data because in both cases the limits of agreement are small and include 0. For respiration frequency, 90.4% of the data are within the limits of agreement, while for TV, 88.2% are within the limits.

The results for breath detection are quite satisfactory; however, the estimation of volumes has some challenges. Among them is the great variability between the morphology of the subjects, the placement of electrodes and the quality of the electrode-skin interface. Those parameters vary among subjects, and these slight differences affect the measurements. These differences make it difficult to generalize a model for all subjects, but a customized analysis allows the estimation of volumes in individual cases. The relationship between the tidal volumes of each subject with the data obtained with the EIT device is evident; however, further analysis of the data is necessary to formulate a complete model.

This first prototype lays the design foundation for a portable EIT device. The system is easy to operate and has a user-friendly form factor with 3D-printed packaging. The implications and applications of the device are mainly oriented to respiratory monitoring in diverse domains: intensive care, geriatric care, home care, medical monitoring, sports, etc.

Although these results are promising, further research is needed to optimize the device for wider clinical use. The next steps include refining the design of the device, making it fully self-contained, improving the reconstruction algorithms, and conducting long-term studies to evaluate its efficacy in relevant environments.

The architecture of the current source, multiplexers, and voltage amplification has many advantages, so no changes are considered. The operation of the multiplexers was the major time addition in the program flow by having to wait for their settling. This is attributed to the models used and can be replaced by more modern analog multiplexer options with lower internal resistances and switching times. One remaining aspect to implement is the battery and dual power module, along with the charging circuitry to make the device completely wireless and portable.

More research is needed in image reconstruction to optimize hyperparameters, models and algorithms for raw image reconstruction. With better fidelity of reconstructed images, ROI segmentation methods can be optimized, thus improving analysis indicators. Future work includes developing a model for a robust estimator for spirometric volumes and defining a calibration procedure for each subject. By addressing these areas, we expect to enhance the capabilities of the device and contribute to the development of comprehensive and personalized healthcare solutions.

To conclude, this work presents the preliminary results of the development of an EIT device, designed and implemented from scratch. After obtaining promising results, such as the correct functioning of the device in human subjects, and the acquisition of signals and images capable of estimating respiratory rate and volume, there is still work to be conducted and things to be improved. We hope that in the near future, this type of development will have a positive impact on society and will contribute to health and well-being, allowing easy and comfortable pulmonary tracking for more personalized medicine.

## Figures and Tables

**Table 1:** Combinations for current multiplexer outputs *A* and *B*, and electrode connections (E).

E Pair	Mux *A*	Mux *B*	E Pair	Mux *A*	Mux *B*
1–2	0	0	9–10	4	4
2–3	0	1	10–11	4	5
3–4	1	1	11–12	5	5
4–5	1	2	12–13	5	6
5–6	2	2	13–14	6	6
6–7	2	3	14–15	6	7
7–8	3	3	15–16	7	7
8–9	3	4	16–1	7	0

**Table 2:** SS11LA flowmeter configuration in Biopac Student Lab PRO Software version 3.7.2.

Digital Filter	Value	Hardware	Value
Filter	1	Sampling frequency	200 S/s
Type	Low-pass	Gain	×5000
Frequency	66.5 Hz	Compensation	0 L/s
Quality factor (Q)	0.5	Input	DC

**Table 3:** Matrices for stimulation patterns 1, 2, 3, 4, 14, 15, and 16.

Electrode Index	*s* *tim_ pattern(1)*	*stim_ pattern(2)*	*stim_ pattern(3)*	*stim_ pattern(4)*	…	*stim_ pattern(14)*	*stim_ pattern(15)*	*stim_ pattern(16)*
1	1	0	0	0	.	0	0	1
2	−1	−1	0	0	.	0	0	0
3	0	1	1	0	.	0	0	0
4	0	0	−1	−1	.	0	0	0
5	0	0	0	1	.	0	0	0
6	0	0	0	0	.	0	0	0
7	0	0	0	0	.	0	0	0
8	0	0	0	0	.	0	0	0
9	0	0	0	0	.	0	0	0
10	0	0	0	0	.	0	0	0
11	0	0	0	0	.	0	0	0
12	0	0	0	0	.	0	0	0
13	0	0	0	0	.	0	0	0
14	0	0	0	0	.	−1	0	0
15	0	0	0	0	.	1	1	0
16	0	0	0	0	.	0	−1	−1

**Table 4:** Anthropometric data of the subjects.

Data	Mean	Std. Dev.	Unit
Age	24.4	2.6	years
Weight	73.5	13.0	kg
Height	169	8	cm
Circ	91	9	cm

**Table 5:** Respiration detection approach comparison.

		Voltage Signal	Pixel Value
Data	*n* *	877	869
Correlation	Slope	0.9901	0.9707
	Intercept	0.0493	0.1588
	Pearson’s *r*	0.9674	0.9205
	Spearman’s ρ	0.9618	0.9284
	RMSE *	0.4662	0.7377
Bland–Altman	Difference STD	0.4663	0.7391
	Difference median	0.0100	0.0050
	RPC *	0.7051	0.8936
	Skewness	−0.3077	1.9937
	Kurtosis	8.5129	21.373

* *n*: quantity, RMSE: root mean square error, RPC: reproducibility coefficient.

**Table 6:** Confusion matrix for breath detection.

		Voltage Signals			Pixel Values		
		**Positives**	**Negatives**	**Total**	**Positives**	**Negatives**	**Total**
Volume	Positives	902	0	902	895	7	902
	Negatives	2	-		5	-	
	Total	904			900		

**Table 7:** Detailed results for TV predictions. Columns shows (1) the identifier of the subject, (2) measurement used for prediction, (3) number of breaths (length of TV array), (4) mean of TV observed from volume signals, (5) mean of TV predicted from voltage signals, (6, 7) slope *b* and intercept *a* coefficients of linear regression y=a+bx used to predict TV, (8, 9, 10) Pearson correlation coefficient *r*, root mean square error and mean absolute percentage error between observed and predicted TV, respectively.

Subject ID	Measurement	Breaths	TV Observed Mean (L)	TV Predicted Mean (L)	Slope	Intercept	Correlation	RMSE (L)	MAPE (%)
S1	16	40	1.314	1.305	79.23	0.37	0.896	0.112	6.89
S2	67	36	0.590	0.587	10.41	0.11	0.730	0.154	22.67
S3	29	21	1.411	1.411	38.79	0.00	0.837	0.234	15.56
S4	155	35	0.850	0.844	7.46	0.22	0.871	0.092	8.41
S5	132	24	1.128	1.104	19.61	0.57	0.818	0.164	10.98
S6	42	40	0.607	0.604	15.26	0.13	0.925	0.061	8.51
S8	181	55	0.501	0.498	8.77	0.19	0.853	0.051	8.23
S9	25	24	1.887	1.855	19.17	0.77	0.819	0.295	12.36
S10	120	79	0.731	0.725	11.63	0.44	0.792	0.081	8.67
S11	16	17	1.531	1.530	25.56	0.01	0.977	0.094	5.13
S12	69	36	0.587	0.577	15.15	0.37	0.814	0.070	9.43
S14	180	38	0.950	0.941	33.24	0.32	0.850	0.097	8.06
S15	144	22	0.842	0.837	5.03	0.11	0.912	0.097	8.75
S16	145	24	1.460	1.456	15.44	0.10	0.950	0.129	8.13
S17	42	20	2.077	2.032	35.24	0.91	0.816	0.356	12.60
S18	167	44	0.704	0.692	13.80	0.49	0.761	0.092	10.38
S19	29	33	1.619	1.615	29.42	0.16	0.895	0.189	9.41
S20	141	51	0.516	0.512	20.64	0.19	0.693	0.079	10.69
S21	83	42	0.731	0.722	77.00	0.36	0.745	0.151	14.65
S22	16	55	0.738	0.730	18.53	0.45	0.780	0.085	8.50
S23	120	28	1.905	1.905	44.37	0.00	0.958	0.124	5.17
S24	194	29	1.179	1.174	12.29	0.14	0.958	0.095	6.15
S25	67	33	0.382	0.374	5.49	0.27	0.527	0.102	19.27
S26	94	41	0.855	0.845	42.51	0.40	0.819	0.116	11.22
S27	140	28	0.647	0.631	45.12	0.46	0.807	0.099	12.84

## Data Availability

The original data presented in the study are openly available in GitHub at https://github.com/fabobug/LIA-v3-Data, accessed on 5 September 2024.

## Abbreviations

The following abbreviations are used in this manuscript:

EIT	Electrical Impedance Tomography
ADC	Analog-to-Digital converter
COPD	Chronic Obstructive Pulmonary Disease
DAC	Digital-to-Analog converter
CW	Cosine wave
OTA	Operational transconductance amplifier
VCCS	Voltage Controlled Current Source
INA	Instrumentation amplifier
CMRR	Common-mode rejection ratio
DRL	Driven Right Leg
DC	Direct current
GND	Ground
PCB	Printed circuit board
meas	Measurements
fps	Frames per second
TV	Tidal volume
RPC	Reproducibility coefficient
IQR	Interquartile range
RMSE	Root mean square error
MAPE	Mean absolute percentage error
SNR	Signal to Noise Ratio
IC	Integrated Circuit
